# GLP-1 Receptor Agonists in Solid Tumour Therapy: Exploring Their Anticancer Potential and Underlying Molecular Pathways

**DOI:** 10.3390/genes16111352

**Published:** 2025-11-10

**Authors:** Daniela Lucente, Stefania Bellino, Anna La Salvia

**Affiliations:** National Center for Drug Research and Evaluation, Istituto Superiore di Sanità, 00161 Rome, Italy; daniela.lucente@iss.it (D.L.); stefania.bellino@iss.it (S.B.)

**Keywords:** GLP-1 receptor agonists, solid tumours, molecular mechanisms, antitumor activity, inflammation and cancer

## Abstract

Glucagon-like peptide-1 receptor agonists (GLP-1 RAs), initially developed to treat type 2 diabetes mellitus, are now being investigated as agents in oncology. Recent preclinical studies have demonstrated their antitumor activity in several solid malignancies, including pancreatic, colorectal, breast, and prostate. Importantly, GLP-1 RAs modulate key signalling pathways such as PI3K/Akt, PKA, and AMPK, and exert anti-inflammatory effects by reducing cytokine production and macrophage infiltration. Preclinical data support their antineoplastic activity in vitro and in vivo, particularly by inhibiting tumour growth and metastasis. Nevertheless, there are ongoing concerns about tumorigenic effects in certain cancer types. This review critically examines the molecular mechanisms by which GLP-1 RAs influence cancer cell proliferation, apoptosis, angiogenesis, and inflammation, and emphasizes the need for further clinical studies to determine their therapeutic relevance. It also proposes assessing GLP-1 RAs as adjuncts in the management of solid tumours.

## 1. Introduction

GLP-1 receptor agonists (GLP-1 RAs) are a class of pharmacological agents that act by mimicking the effects of the endogenous incretin hormone GLP-1 (glucagon-like peptide-1). They are primarily prescribed for the management of type 2 diabetes mellitus (T2DM) and, more recently, for obesity. By binding to GLP-1 receptors, these agents enhance glucose-dependent insulin secretion, suppress glucagon release, slow gastric emptying, and reduce appetite, which collectively contribute to improved glycaemic control and weight loss [1]. Given their role in influencing metabolic status, body weight, and inflammatory pathways, there is growing interest in the potential impact of GLP-1 RAs on cancer risk [2]. Research is ongoing to determine whether these agents have a protective, neutral, or adverse effect on the development and progression of various types of cancer.

The treatment of solid tumours, including those arising in the lung, colorectum, breast, and pancreas, remains an open challenge in contemporary oncology. Despite the implementation of multimodal therapeutic strategies, comprising surgical resection, cytotoxic chemotherapy, radiotherapy, targeted molecular therapies, and, more recently, immunotherapy, the overall prognosis for many solid malignancies remains unsatisfactory, particularly in advanced or metastatic stages. One of the most critical barriers to effective treatment is the emergence of drug resistance, both intrinsic and acquired, which compromises the long-term efficacy of systemic therapies [3]. Another major complicating factor is the profound intra- and inter-tumoural heterogeneity observed across solid tumours, encompassing differences in genetic profiles, epigenetic modifications, metabolic states, and interactions with the tumour microenvironment (TME). Additionally, the capacity of tumour cells to undergo epithelial–mesenchymal transition facilitates metastatic dissemination, enabling the colonization of distant organs and the establishment of secondary lesions that are often more resistant to conventional treatments [4]. Taken together, these multifaceted and interrelated mechanisms underline the complexity of solid tumour biology and highlight the urgent need for novel therapeutic approaches. Such strategies should ideally be capable of overcoming drug resistance, selectively targeting tumour-specific molecular pathways, modulating the TME, and enhancing the efficacy of existing treatment modalities.

In this context, the exploration of innovative pharmacological agents, including those originally developed for non-oncological indications, is gaining increasing attention and may pave the way for the development of more effective and individualized cancer therapies [5].

This review explores the molecular mechanisms, preclinical findings, and emerging clinical data regarding the potential utility of GLP-1 RAs in the treatment of solid cancers. In this review, we propose a conceptual framework in which the pleiotropic actions of GLP-1 Ras, particularly their metabolic, anti-inflammatory, and signalling-modulatory properties, may be leveraged to address critical barriers in solid tumour treatment. These include the modulation of the TME, interference with resistance-associated signalling pathways (e.g., phosphatidylinositol 3-kinase PI3K/Akt), and possible synergistic effects with chemotherapy. By reframing GLP-1 RAs in this context, we aim to provide a novel interpretation of their potential role in oncology. In addition, possible risks, benefits, and areas for further research to establish the role of these agents in modern oncology will be discussed.

## 2. GLP-1 Receptor Agonists

GLP-1 plays a central role in maintaining glucose homeostasis by stimulating insulin release from pancreatic β-cells in a glucose-dependent manner, while simultaneously suppressing the secretion of glucagon from α-cells, thereby reducing hepatic glucose production. Furthermore, GLP-1 slows gastric emptying and promotes satiety through central mechanisms, contributing to reduced food intake and subsequent weight loss—effects that are particularly beneficial in overweight or obese patients with T2DM [6]. GLP-1 RAs exert their effects by binding to and activating the GLP-1 receptor, a G protein-coupled receptor expressed primarily in pancreatic islets but also in various extrapancreatic tissues, including the gastrointestinal tract, heart, brain, and kidneys. This widespread receptor distribution explains the broad range of metabolic and potentially pleiotropic effects of these agents [7].

Over the past two decades, GLP-1 RAs have become a cornerstone in the treatment algorithm for T2DM, especially in patients who require improved glycaemic control with the added benefit of weight reduction and cardiovascular protection. Their glucose-lowering efficacy, low risk of hypoglycaemia (due to their glucose-dependent mechanism of action), and favourable impact on cardiovascular outcomes have led to their widespread adoption in clinical practice.

To date, six GLP-1 RAs have been approved in the European Union for the treatment of T2DM (Table 1). Exenatide, derived from Gila monster saliva, was the first GLP-1 RA, initially given twice daily and later as a once-weekly formulation [8]. Liraglutide, a once-daily GLP-1 RA, provides glycaemic control and weight loss, while lixisenatide mainly reduces postprandial glucose [9]. Liraglutide has also been combined with insulin degludec in a fixed-ratio formulation [10]. Dulaglutide is a once-weekly GLP-1 RA with prolonged activity due to IgG4 Fc fusion. Semaglutide, another long-acting GLP-1 RA with high receptor affinity, enables once-weekly dosing and shows superior glucose and weight outcomes [11]. The most recent addition to the class is tirzepatide.

A growing body of preclinical evidence, primarily derived from in vitro studies, has highlighted the potential anticancer properties of GLP-1 RAs. These agents, originally developed for glycaemic control in T2DM, have demonstrated promising effects on various cancer-related cellular processes, including the inhibition of tumour cell proliferation, the induction of programmed cell death (apoptosis), and the modulation of angiogenesis—the process by which new blood vessels are formed to supply nutrients to tumour tissues [12]. These biological effects suggest that GLP-1 RAs may exert direct and indirect antitumour activity across several solid tumour types.

Experimental data have suggested beneficial effects of GLP-1 RAs in colorectal, prostate, gall bladder, ovarian, and endometrial cancers, with studies reporting reduced tumour cell viability, impaired metastatic potential, and enhanced sensitivity to other therapeutic agents. These findings are largely based on cellular and animal models, where GLP-1 RAs modulate key intracellular pathways such as PI3K/Akt, AMPK, and PKA, contributing to decreased cell proliferation and increased apoptotic signalling [13]. In some cases, these agents have also shown potential to interfere with tumour angiogenesis, a critical factor in tumour progression and metastasis, through inhibition of pro-angiogenic factors or signalling cascades [14].

However, despite these encouraging preclinical outcomes, concerns have been raised regarding the potential tumorigenic risks associated with GLP-1 receptor activation in certain contexts. For example, in some preclinical and observational studies, liraglutide, one of the most widely used GLP-1 RAs, was associated with an increased incidence of thyroid C-cell hyperplasia and carcinomas, particularly in rodent models, where GLP-1 receptors are more abundantly expressed in thyroid tissue than in humans [15]. Additionally, isolated reports have suggested a possible link between liraglutide and elevated risks of breast and pancreatic cancers, although these findings remain controversial and have not been confirmed in large-scale clinical trials. Regulatory agencies, including the European Medicines Agency (EMA) and US Food and Drug Administration (FDA), have acknowledged these potential signals but have not found conclusive evidence warranting withdrawal or restriction of GLP-1 RAs, emphasizing the need for long-term pharmacovigilance and focused research.

Preliminary evidence suggests that GLP-1 RAs, such as exendin-4, can enhance the effectiveness of chemotherapies like gemcitabine in pancreatic cancer, reducing tumour burden more than either treatment alone through complementary antiproliferative and pro-death mechanisms [16].

The favourable safety profile and the pleiotropic metabolic effects of GLP-1 RAs, such as weight reduction, anti-inflammatory activity, and improved insulin sensitivity, further support their potential utility in oncology [17]. These characteristics could offer added benefit in patients with metabolic syndrome, obesity, or insulin resistance—conditions that are increasingly recognized as risk factors for cancer development and progression. Nevertheless, the translation of preclinical findings into clinical practice remains limited. Currently, there are no approved oncological indications for GLP-1 RAs, and clinical studies exploring their anticancer potential are scarce. Major barriers include a lack of awareness among oncologists and clinical researchers, limited data from human studies, and the complexity of defining the precise mechanisms of action in different tumour types [18].

Table 1 includes GLP-1 RAs approved in the European Union for the treatment of T2DM. Table 2 represents the effects and mechanisms of GLP-1 RAs in solid tumours.

## 3. Materials and Methods

A comprehensive literature search was performed using major biomedical databases, including PubMed, Scopus, and Web of Science, covering publications up to September 2025. The following keywords and combinations were used: “GLP-1 receptor agonists”, “solid tumours”, “cancer”, “proliferation”, “apoptosis”, “angiogenesis”, “inflammation”, “PI3K/Akt”, “molecular mechanisms”. Original research articles, preclinical studies, and relevant reviews written in English were included. Particular emphasis was placed on studies elucidating the molecular pathways modulated by GLP-1 RAs and their biological effects in cancer models. Articles were selected based on relevance, methodological rigor, and contribution to the understanding of GLP-1 RAs in oncology. Data were extracted, synthesized, and organized according to thematic areas, including mechanisms of action and tumour-specific effects. No formal quality assessment or meta-analysis was performed, in line with the scope of a narrative review.

## 4. Molecular Mechanisms of GLP-1 Receptor Agonists in Cancer

### 4.1. Influences on Cellular Proliferation, Apoptosis, and Angiogenesis

GLP-1, traditionally known for its role in metabolic regulation, also functions as a growth factor that influences cell proliferation, differentiation, and survival across various tissues. Acting through its receptor (GLP-1R) and associated signalling pathways, it enhances cell viability and proliferation while suppressing apoptosis [19]. GLP-1 promotes cell proliferation mainly through activation of the phosphatidylinositol 3-kinase (PI3K)/Akt signalling pathway, which regulates cell growth, metabolism, and survival. When GLP-1 binds to its receptor, it triggers PI3K activation and subsequent Akt phosphorylation, leading to the regulation of transcription factors and targets that drive cell cycle progression and prevent apoptosis [20]. In addition, GLP-1 influences key transcription factors such as PDX1 and FoxO1 through the PI3K/Akt pathway. It enhances PDX1 expression and nuclear localization, which supports β-cell identity, promotes proliferation, and boosts insulin production—processes essential for maintaining β-cell function and compensating for insulin resistance [21,22]. In parallel, GLP-1 signalling through the PI3K/Akt pathway inactivates the transcription factor FoxO1, preventing it from suppressing genes that promote cell growth and survival. This enhances β-cell proliferation and viability. Since GLP-1 receptors are also present in other tissues—including the heart, brain, gut, and certain tumours—their proliferative effects may extend beyond pancreatic cells, highlighting possible implications for cancer biology [23]. Overall, the role of GLP-1 as a modulator of cell proliferation is mediated by a network of tightly regulated intracellular mechanisms, with the PI3K/Akt-PDX1-FoxO1 axis playing a pivotal role. These interactions underscore the complexity of GLP-1’s biological actions and highlight the importance of context-dependent signalling, which may yield either beneficial or deleterious effects depending on the tissue type, receptor distribution, and cellular environment [24].

Numerous experimental and preclinical studies have investigated the potential anticancer effects of GLP-1 RAs, revealing their capacity to interfere with key biological processes involved in tumour development and progression. Among the most notable findings is their ability to inhibit cellular proliferation in various cancer models. In vitro and in vivo data have shown that GLP-1 RAs can reduce the proliferative capacity of a wide range of tumour cell types, including breast, prostate, ovarian, and pancreatic cancers [25,26]. GLP-1 RAs can inhibit cell proliferation by modulating signalling pathways that control the cell cycle and mitosis, suppressing oncogenic pathways like MAPK/ERK and activating tumour-suppressive ones such as AMPK and p53. They also influence apoptosis, promoting or preventing programmed cell death depending on the tissue context, thus playing a dual and context-dependent role in regulating cell survival [20]. In pancreatic β-cells, GLP-1 increases the ATP/AMP ratio, a key indicator of cellular energy status, leading to activation of AMP-activated protein kinase (AMPK), which is known to participate in the regulation of apoptosis and metabolic stress responses [27]. In these cells, GLP-1-induced AMPK activation is associated with enhanced β-cell survival under hyperglycaemic conditions, contributing to the preservation of pancreatic function in diabetic patients. Furthermore, GLP-1 can engage the protein kinase A (PKA)-dependent pathway, which not only supports insulin secretion but also promotes the expression and activity of pancreatic duodenal homeobox-1 (PDX1)—a key transcription factor involved in pancreatic development and β-cell survival. Through PKA-mediated phosphorylation events, GLP-1 signalling enhances the nuclear translocation and DNA-binding ability of PDX1, ultimately fostering cell viability and resistance to apoptosis. In cardiomyocytes, GLP-1 activates the PI3K/Akt pathway, which inhibits pro-apoptotic factors (Bad, GSK-3β) and promotes anti-apoptotic proteins (Bcl-2), reducing cell death during stress like ischemia-reperfusion. This highlights GLP-1’s tissue-specific, cardioprotective effects [28]. Additionally, GLP-1 inhibits the activity of caspase-3, a critical executioner enzyme in the apoptotic cascade that mediates DNA fragmentation and dismantling of cellular components. By suppressing caspase-3 activity, GLP-1 can prevent premature or pathological cell death in certain contexts, further demonstrating its cytoprotective potential [27].

The mechanistic underpinnings of GLP-1-induced angiogenesis are multifactorial and involve the activation of several crucial intracellular signalling pathways that regulate endothelial cell behaviour. Among these, the PI3K/Akt pathway plays a central role. Activation of PI3K leads to the phosphorylation and subsequent activation of Akt, which promotes endothelial cell survival, nitric oxide production, and cytoskeletal rearrangement—all vital for angiogenic processes [29]. In addition, the Src family kinases, a group of non-receptor tyrosine kinases, are also engaged upon GLP-1 receptor stimulation. Src kinases facilitate cytoskeletal remodelling and promote the assembly of focal adhesions, further enhancing the motility and invasiveness of endothelial cells during vessel sprouting. Moreover, the PKA pathway is involved in mediating GLP-1’s angiogenic effects. PKA, activated via cyclic AMP (cAMP) signalling, modulates various downstream effectors that contribute to endothelial cell proliferation and migration. The coordinated activation of PI3K/Akt, Src, and PKA pathways orchestrates a complex network of intracellular events that culminate in the promotion of angiogenesis.

These findings have important implications not only for understanding the physiological roles of GLP-1 but also for exploring its potential therapeutic applications. Given that angiogenesis is a critical process in tissue repair, wound healing, and ischemic disease, GLP-1 or its analogues might be harnessed to promote vascular regeneration in clinical settings. Conversely, in the context of cancer, these pro-angiogenic effects of GLP-1 RAs represent a potential risk rather than an unequivocal benefit. In the setting of solid tumours, neovascularisation can facilitate tumour growth and metastasis by improving perfusion, nutrient supply, and escape routes for malignant cells. Therefore, the use of GLP-1 RAs in a cancer context demands careful contextual assessment. Strategies to mitigate this risk may include selecting tumour types with low angiogenic dependency, monitoring tumour vascular markers before and during treatment, and combining GLP-1 RAs with anti-angiogenic therapies to offset potential pro-vascularisation. In addition, mechanistic studies should assess whether GLP-1-driven angiogenesis in the tumour microenvironment is qualitatively different from physiological angiogenesis (for example, by examining differences in vessel maturity, pericyte coverage, and hypoxia-driven signalling). Such a nuanced approach enables the integration of GLP-1 RAs’ pleiotropic effects into oncologic regimens while minimising unintended promotion of tumour vasculature [30].

### 4.2. Anti-Inflammatory Effects

Chronic inflammation is widely recognized as a critical and well-established factor in the progression and development of various types of cancer, playing a particularly significant role in malignancies such as colorectal cancer (CRC) and pancreatic cancer (PDAC). The persistent presence of inflammatory mediators within the TME fosters conditions that promote genetic mutations, cellular proliferation, angiogenesis, and metastasis, all of which contribute to the aggressive nature of these cancers. Chronic inflammation creates a sustained state of oxidative stress and DNA damage, thereby facilitating oncogenesis and tumour progression [31]. In this context, GLP-1 has anti-inflammatory properties, particularly by modulating inflammatory processes associated with innate immune cells such as macrophages. Macrophages are key players in the inflammatory cascade and TME, often contributing to the secretion of pro-inflammatory cytokines that exacerbate disease progression. One of the critical pathways involved in macrophage-mediated inflammation is the nuclear factor kappa-light-chain-enhancer of activated B cells (NF-κB) pathway, which regulates the transcription of multiple inflammatory genes [32].

A notable study employed GLP-1-RAs to investigate the anti-inflammatory effects of GLP-1 signalling on macrophages. Utilizing the macrophage cell line RAW264 and in vivo models, their findings demonstrated a significant reduction in macrophage infiltration into tissues, along with a pronounced suppression of the release of key pro-inflammatory cytokines, including tumour necrosis factor-beta (TNF-β), interleukin-6 (IL-6), and interleukin-1 beta (IL-1β) [31,33]. These cytokines are known to play pivotal roles in sustaining chronic inflammatory states and are heavily implicated in the pathogenesis of insulin resistance—a metabolic disorder strongly linked to the development of T2DM and a heightened risk of cancer.

Importantly, the anti-inflammatory actions of GLP-1-RAs have been hypothesized to contribute not only to the mitigation but potentially to the complete inhibition of insulin resistance. Insulin resistance itself is characterized by a diminished cellular response to insulin, leading to hyperinsulinemia and a metabolic milieu conducive to tumorigenesis. Chronic insulin resistance promotes increased production of reactive oxygen species (ROS), which cause oxidative damage to DNA and proteins, facilitating mutagenesis and carcinogenesis. By reducing inflammation and oxidative stress, GLP-1 may therefore exert a protective effect against the initiation and progression of malignancies in patients with insulin resistance [34].

Given the well-documented association between chronic inflammation, insulin resistance, and cancer risk, GLP-1’s capacity to modulate inflammatory pathways positions it as a promising molecule with potential oncoprotective properties. The ability of GLP-1 to inhibit macrophage-driven inflammation and cytokine release suggests a mechanistic basis through which it may interfere with the tumour-promoting microenvironment. This regulatory effect on inflammation not only has implications for metabolic diseases but also raises the possibility that GLP-1 receptor agonists could be harnessed as adjunct therapies to reduce cancer risk or progression, especially in inflammation-associated cancers such as colorectal and pancreatic carcinomas [35].

In CRC, chronic intestinal inflammation has been closely linked to increased tumorigenesis and progression. Tumour-associated macrophages (TAMs) contribute to the secretion of pro-tumorigenic cytokines and facilitate immune evasion, angiogenesis, and metastatic spread. GLP-1 RAs have been shown to reduce the recruitment and activation of inflammatory macrophages and to downregulate NF-κB signalling, a pathway central to both inflammatory bowel pathology and CRC progression. By attenuating this axis, GLP-1 RAs may disrupt the pro-inflammatory feedback loop that sustains tumour growth and resistance to therapy in CRC [36].

Similarly, PDAC is characterized by a profoundly immunosuppressive and desmoplastic TME, in which chronic pancreatitis and macrophage-mediated inflammation play key roles in tumour initiation and progression. GLP-1 RAs have demonstrated anti-inflammatory properties in pancreatic islet inflammation, including the ability to suppress the expression of pro-inflammatory cytokines in macrophages and ductal epithelial cells. These effects may translate into a dampening of the inflammatory milieu that fosters immune tolerance and stromal activation in PDAC [37].

Moreover, GLP-1 RAs may promote a shift in macrophage polarization from the M2 to the M1 phenotype, thereby restoring antitumour immune responses and enhancing the potential efficacy of immune checkpoint blockade, an area of growing interest in both CRC and PDAC, where response rates to immunotherapy remain modest.

Taken together, these findings support the hypothesis that GLP-1-based therapies could modulate key inflammatory and immune mechanisms underlying inflammation-associated cancers (e.g., CRC and PDAC), potentially reducing progression or improving responsiveness to existing treatments [38].

Further research is warranted to elucidate the precise molecular interactions by which GLP-1 modulates immune responses in the TME, as well as to determine the clinical relevance of these anti-inflammatory effects in cancer prevention and therapy.

**Figure 1:**
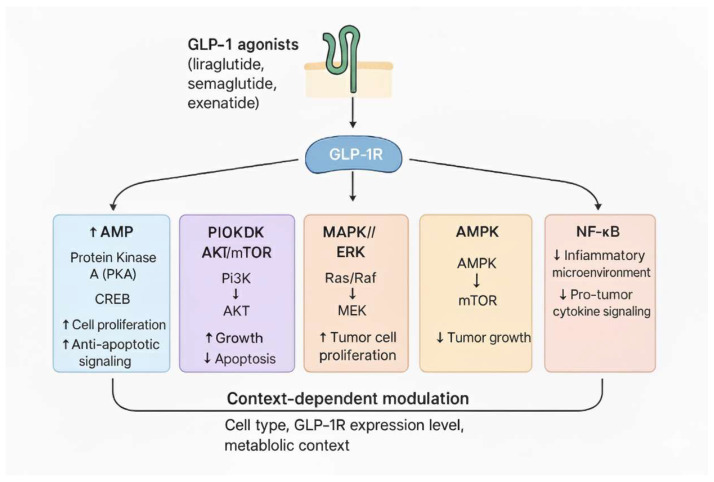
Pathways activated by GLP-1R and their potential influence on tumour growth. PKA, protein kinase A; CREB, cAMP response element-binding protein; PIOKDK, phosphoinositide-dependent protein kinase; AKT, protein kinase B; mTOR, mammalian target of rapamycin; MAPK, mitogen-activated protein kinase; ERK, extracellular signal-regulated kinase; AMPK, AMP-activated protein kinase; NK-kB, nuclear factor kappa-light-chain-enhancer of activated B cells.

Figure 1 describes the pathways activated by GLP-1R and their potential influence on tumour growth.

## 5. Role of GLP-1 Agonists in Cancer: Preclinical Evidence

While the definitive role of GLP-1 RAs in oncological treatment is still under active investigation, an increasing body of recent preclinical evidence suggests that these agents may possess significant anticancer properties, potentially expanding their therapeutic applications beyond their established use in metabolic disorders such as T2DM. Although their incorporation into standard cancer therapy protocols remains in the exploratory phase, early experimental findings offer encouraging insights into their potential to modulate tumour biology through multiple mechanisms.

Collectively, these findings suggest that GLP-1 RAs exhibit strong antitumor effects in pancreatic, colorectal, breast, and prostate cancer types, primarily via modulating key signalling pathways such as PI3K/Akt, PKA, and AMPK, and exerting anti-inflammatory effects [39].

A growing number of in vitro and in vivo preclinical studies have demonstrated the capacity of GLP-1 RAs to inhibit key hallmarks of cancer, including tumour cell proliferation, migration, invasion, and metastatic potential. Previous studies provided robust evidence of the antitumor activity of liraglutide, a long-acting GLP-1 analogue, in which liraglutide was shown to significantly reduce both the tumorigenicity and metastatic spread of human pancreatic cancer cells [40]. This effect was observed not only in vitro but also in murine xenograft models of human pancreatic carcinoma, where liraglutide treatment led to a notable suppression of tumour growth and metastatic progression.

Mechanistically, the antineoplastic effect of liraglutide was attributed to the inhibition of the PI3K/Akt signalling pathway, a critical molecular cascade involved in regulating cell survival, proliferation, and motility. Aberrant activation of PI3K/Akt is commonly observed in various malignancies, and its suppression by GLP-1R agonism suggests a plausible therapeutic mechanism by which tumour progression could be attenuated. The study thus underscored the potential of liraglutide as a modulator of intracellular oncogenic signalling networks [41].

The coexistence of physiological pro-proliferative effects (e.g., in pancreatic β-cells) and direct antitumour effects in cancer cells represents the central conceptual dilemma for the clinical translation of GLP-1 RAs. To reconcile this apparent contradiction, an analysis based on tissue specificity, receptor expression levels, and the contextual regulation of the PI3K/Akt pathway is essential. In non-malignant cells (such as β-cells), GLP-1R activation promotes survival through the PI3K/Akt pathway and the PDX1-FoxO1 axis, in a mechanism that is strictly regulated and glucose-dependent. Conversely, in many solid tumours (e.g., pancreas, colorectum), the PI3K/Akt signal is often hyperactivated due to intrinsic oncogenic mutations. It is hypothesized that GLP-1 RAs act here as modulators, exerting a negative feedback inhibition on the hyperactive PI3K/Akt signal, or by diverting receptor activation toward other pathways like AMPK, which induces cell cycle arrest and apoptosis in tumour contexts. Furthermore, the type of analogue (e.g., liraglutide vs. exenatide) and its different pharmacokinetics and receptor affinities might influence signal selectivity and the duration of exposure, contributing to the variations observed between studies [42].

Another important contribution to the field comes from a work that explored the role of GLP-1 RAs in intrahepatic cholangiocarcinoma (iCCA), a rare but aggressive malignancy of the bile ducts. Using two iCCA cell lines, KKU-055 and KKU-213A, the authors evaluated the effects of exendin-4 and liraglutide on tumour cell behaviour, particularly in relation to cell proliferation and migratory capacity. While neither agent significantly inhibited cellular proliferation in vitro, liraglutide induced a substantial reduction in cell migration, which was associated with the downregulation of pathways involved in the EMT [43]. Interestingly, the in vivo component of the study yielded even more compelling evidence. In murine xenograft models of iCCA, liraglutide administration led to a significant decrease in tumour volume and weight, despite its minimal effect on proliferation in vitro. This discrepancy suggests that the drug’s antitumour efficacy may rely on mechanisms that operate specifically in the in vivo milieu, rather than direct cytotoxicity in isolated tumour cells. Several non-mutually exclusive mechanistic hypotheses may account for the observed discrepancy between the in vitro and in vivo effects of liraglutide. One plausible explanation involves the drug’s immunomodulatory properties: liraglutide may influence macrophage infiltration and polarization within the tumour microenvironment, thereby attenuating pro-tumoral inflammation while promoting more effective antitumour immune surveillance [44]. Additionally, liraglutide may exert anti-angiogenic effects, contributing to vascular remodelling that enhances tumour perfusion and reduces hypoxia-induced resistance mechanisms [45]. A further possibility is that liraglutide mediates stromal reprogramming, including the modulation of fibroblast activity and remodelling of the extracellular matrix, which could facilitate improved drug penetration and alter tumour–stroma interactions in a manner that impairs tumour progression. Together, these mechanisms underscore the importance of the tumour microenvironment in shaping the therapeutic response to GLP-1 RAs and may explain the greater efficacy observed in vivo models compared to isolated in vitro systems. Furthermore, the study reported downregulation of GLP-1R expression following liraglutide treatment in both in vitro and in vivo settings, suggesting a feedback regulatory mechanism that may modulate drug sensitivity or resistance over time.

Taken together, these findings support the hypothesis that GLP-1-based therapies may offer therapeutic benefit not only for metabolic regulation in patients with T2DM but also as adjunctive or supportive agents in oncology, particularly in cancers of the pancreas and liver. Importantly, these antitumor effects appear to be context-dependent, varying according to tumour type, receptor expression levels, and the specific GLP-1 RA employed.

Although more comprehensive clinical trials are necessary to establish safety, efficacy, and optimal dosing strategies in oncological settings, the data thus far indicate that GLP-1 receptor agonists do not exacerbate tumour growth and may counteract it through multiple molecular mechanisms. This opens the door to further research into repositioning of GLP-1 RAs as multi-functional agents capable of addressing both metabolic and oncologic disease burdens in an integrated manner.

## 6. Discussion

The potential role of GLP-1 RAs in the treatment of solid tumours represents a compelling and rapidly developing field within translational oncology. Originally designed for the management of T2DM, GLP-1 RAs are now garnering attention for their pleiotropic effects, particularly their ability to influence cellular processes relevant to tumour biology.

Accumulating preclinical evidence demonstrates that these agents may exert antiproliferative, pro-apoptotic, anti-inflammatory, and anti-angiogenic effects across a range of malignancies, including PDAC, CRC, breast, and prostate cancers.

Of note, the molecular and systemic effects of GLP-1 RAs may intersect with core biological mechanisms that contribute to the development of the inefficacy of anticancer treatment in solid tumours. Among these mechanisms, chronic inflammation, metabolic dysregulation, and immunosuppressive remodelling of the TME have emerged as key hallmarks of cancer progression and treatment resistance.

Chronic inflammation plays a central role in shaping a tumour-promoting milieu, sustaining proliferative signalling, evading immune surveillance, and facilitating metastatic dissemination. GLP-1 RAs have been shown to exert potent anti-inflammatory actions across several disease models, mediated through the inhibition of NF-κB signalling, suppression of pro-inflammatory cytokine production (e.g., TNF-α, IL-1β, and IL-6), and ROS production [46]. These anti-inflammatory effects may not only mitigate tumour-associated inflammation but also restore immunological tone within the TME, potentially enhancing the efficacy of immune-based therapies or sensitizing tumours to cytotoxic agents.

Metabolic dysregulation, particularly insulin resistance, hyperinsulinemia, and altered lipid metabolism, is increasingly recognized as a driver of tumour aggressiveness and drug resistance. GLP-1 RAs improve systemic glucose homeostasis and reduce circulating insulin levels, potentially disrupting insulin/IGF-1 signalling pathways frequently co-opted by tumour cells [47]. In addition, GLP-1 RAs influence intracellular energy sensors such as AMPK and inhibit oncogenic pathways including PI3K/Akt/mTOR, which are often implicated in metabolic adaptation and chemoresistance. These properties suggest a potential role for GLP-1 RAs in reprogramming the metabolic landscape of tumours, thereby impairing their survival under therapeutic pressure.

TME represents a dynamic and complex ecosystem composed of stromal cells, immune infiltrates, extracellular matrix components, and vascular networks, all of which contribute to tumour survival and therapy resistance. GLP-1 RAs have demonstrated pleiotropic effects on multiple components of the TME. For instance, they can modulate endothelial function, reduce oxidative stress, and promote vascular normalization, which may enhance drug delivery and reduce hypoxia-induced resistance. Furthermore, GLP-1 RAs have shown anti-fibrotic effects in non-cancer contexts, such as liver and kidney fibrosis, raising the hypothesis that they might remodel desmoplastic stroma and improve therapeutic penetration in solid tumours. Their ability to modulate macrophage polarization and T-cell infiltration further underscores their potential as immunomodulatory agents capable of reversing local immunosuppression [48].

Despite these promising findings, the current evidence is predominantly derived from in vitro experiments and animal models, while clinical data remain limited. Specifically, their anti-inflammatory, metabolic, and microenvironmental effects suggest that GLP-1 RAs may offer therapeutic value beyond glycaemic control, potentially serving as adjunctive agents in cancer therapy.

Therefore, while the biological plausibility of GLP-1 RAs as adjuncts in cancer therapy is strong, especially considering their impact on key molecular pathways such as PI3K/Akt, AMPK, and PKA, the next essential step involves translating these mechanistic insights into well-designed clinical trials. We propose initiating early phase (Phase I/II) trials focusing on inflammation- and metabolism-associated solid tumours, such as CRC and PDAC, where GLP-1 RAs may exert the greatest benefit.

Candidate agents could include liraglutide or semaglutide, given their established safety profiles in non-cancer populations and emerging preclinical evidence of anticancer activity. Careful patient selection should prioritize individuals with metabolic dysregulation, obesity, or insulin resistance, as these factors may predict enhanced therapeutic response [44].

Key endpoints should extend beyond traditional oncologic outcomes (e.g., tumour response, progression-free survival, and overall survival) to include biomarkers of metabolic and inflammatory reprogramming, tumour microenvironment remodelling, and safety in non-diabetic cohorts. Combination strategies, such as co-administration with standard-of-care oncologic treatments including chemotherapy, targeted therapies, and immune checkpoint inhibitors, should also be explored, as GLP-1 RAs may modulate treatment sensitivity through multiple biological axes, potentially offering synergistic effects, potentially enhancing efficacy while mitigating systemic toxicity through their known metabolic benefits [49].

In parallel, window-of-opportunity studies and neoadjuvant settings may provide valuable mechanistic insight, enabling pre- and post-treatment tissue analyses to assess pharmacodynamic changes within the tumour and its microenvironment [50].

Long-term safety will be critical to determining their clinical applicability, particularly in non-diabetic cancer populations where the chronic use of GLP-1 RAs may carry unforeseen risks. Careful assessment of potential adverse events, including pancreatic, thyroidal, and cardiovascular effects, as well as their interactions with standard oncologic therapies, will be essential to ensure that any therapeutic benefit is not offset by toxicity or disease exacerbation over time [38].

An important area for future investigation involves the identification of specific tumour types and molecular subtypes that are most likely to respond to GLP-1 RA treatment. For instance, tumours with high GLP-1R expression or those characterized by dysregulation of insulin/IGF signalling pathways may represent more responsive targets.

Collectively, these targeted trial strategies will be critical to determining the clinical utility and scope of GLP-1 RAs in oncology, moving the field beyond hypothesis and into actionable translational research.

However, despite these promising findings, several key barriers must be addressed before clinical translation of GLP-1 RAs into oncology can be considered viable. First, solid tumours often present unique challenges to drug penetration due to aberrant vascular architecture, elevated interstitial fluid pressure, and dense stromal fibrosis. These factors limit the bioavailability of systemically administered agents within the tumour core. As such, it is crucial to assess whether GLP-1 RAs can effectively reach and accumulate in relevant tumour compartments, especially given the variable expression of GLP-1Rs across different cancer types and within the TME. Second, safety and tolerability in non-diabetic populations remain underexplored. Although GLP-1 RAs are widely used in the management of metabolic diseases, their long-term effects in oncologic patients without pre-existing metabolic dysfunction are not well characterized. This raises important concerns regarding off-target effects, the risk of endocrine disruption, and the potential for adverse events such as pancreatitis or gastrointestinal toxicity [51,52]. Third, the integration of GLP-1 RAs with existing oncologic therapies must be carefully considered. Potential drug–drug interactions, overlapping toxicities, and immunomodulatory effects could influence the efficacy or tolerability of standard treatments, including chemotherapy, targeted therapies, and immune checkpoint inhibitors. Understanding these interactions will be essential for the design of rational and safe combination regimens.

Overall, caution is warranted, particularly regarding the long-term safety profile of GLP-1 RAs in oncologic populations. Some concerns have been raised in the literature, especially related to a possible increased risk of thyroid C-cell hyperplasia and neoplasia, as well as associations with PDAC and breast tumours [53].

These potential risks must be rigorously addressed through long-term observational studies and post-marketing surveillance, especially if these agents are to be repurposed for cancer indications.

## 7. Conclusions

In summary, GLP-1 RAs represent a promising new class of agents that may offer both metabolic and antitumor benefits. Their favourable safety profile in diabetic populations, along with emerging preclinical oncologic evidence, provides a strong rationale for further exploration. However, their use in cancer care must be approached with scientific rigor and clinical caution, balancing enthusiasm for innovation with the need for robust evidence, considering also that their clinical applicability remains contingent upon the resolution of key translational challenges. The future of GLP-1 RAs in oncology will depend on well-designed clinical studies, biomarker-driven patient selection, and a better understanding of their tumour-specific effects and mechanistic pathways. If these hurdles are addressed, GLP-1 RAs may indeed become valuable tools in the evolving landscape of personalized cancer therapy.

## Figures and Tables

**Table 1:** GLP-1 RAs approved in the European Union for the treatment of type 2 diabetes.

Drug Name	Brand Name	Year of Approval
Exenatide	Beyetta	2009
Liraglutide	Victoza	2009
Lixisenatide	Lyxumia	2013
Dulaglutide	Trulicity	2014
Semaglutide	Ozempic	2018
Tirzepatide	Mounjaro	2024

**Table 2:** Effects and mechanisms of GLP-1 RAs in solid tumours.

Category	GLP-1 RA Mechanism/Effect	Key Pathways and Details	Associated Cancer Types/Preclinical Models
Antitumor Activity	Inhibition of Cell Proliferation	Modulates oncogenic (MAPK/ERK) and tumour-suppressive (AMPK, p53) signals.	Breast, Prostate, Ovarian, Pancreatic, Colorectal, etc.
	Inhibition of Growth & Metastasis	Reduces tumorigenicity and metastatic spread by inhibiting pathways like EMT.	Pancreatic Carcinoma (Liraglutide); iCCA (Liraglutide).
	Induction of Apoptosis	Modulates apoptotic cascades, including Caspase-3 activity.	Indicated as a modulated process.
	Synergy with Chemotherapy	Enhances efficacy when combined with standard agents (e.g., gemcitabine).	Pancreatic Carcinoma (Exendin-4 + Gemcitabine).
Molecular Signalling	PI3K/Akt Modulation	Inhibition of this crucial survival pathway in cancer cells.	Proposed mechanism for effect in Pancreatic Carcinoma.
	PKA and AMPK Modulation	Involved in regulating cell survival and metabolic stress response.	General mechanism of action.
Anti-Inflammatory Effects	Suppression of Cytokine Release	Reduces pro-inflammatory mediators (TNF-β, IL-6, IL-1\beta).	Cancers linked to inflammation (e.g., Colorectal, Pancreatic).
	Inhibition of Macrophage Infiltration	Reduces immune cells that promote the pro-tumour microenvironment.	General anti-inflammatory effect.

## Data Availability

No new data were created or analyzed in this study. Data sharing is not applicable to this article.
